# Family physicians' experiences when collaborating with district nurses in home care-based medical treatment. A grounded theory study

**DOI:** 10.1186/1471-2296-11-82

**Published:** 2010-10-27

**Authors:** Sonja Modin, Lena Törnkvist, Anna-Karin Furhoff, Ingrid Hylander

**Affiliations:** 1 Department of Neurobiology, Care Science and Society, Centre for Family and Community Medicine (CeFAM), Karolinska Institutet, Alfred Nobels allé 12, S-14284 Huddinge, Sweden

## Abstract

**Background:**

This article concerns Swedish family physicians' (FPs) experiences collaborating with district nurses (DNs) when the DNs provide medical treatment for home care patients. The aim was to develop a model to illuminate this process from the FPs' perspective.

**Methods:**

Semi-structured interviews were conducted with 13 FPs concerning one of their patients with home care by a DN. The interview focused on one patient's treatment and care by different care providers and the collaboration among them. Grounded theory methodology (GTM) was used in the analyses.

**Results:**

It was essential for FPs to collaborate with and rely on DNs in the medical treatment of home care patients. According to the FPs, factors such as the disease, FPs' working conditions and attitude determined how much of the initiative in this treatment FPs retained or left to DNs. Depending on the circumstances, two different roles were adopted by the individual FPs: *medical conductors *who retain the initiative and *medical consultants *who leave the initiative to DNs. Factors as the disease, DNs' attitudes towards collaboration and DNs' working conditions influenced whether or not the FPs felt that grounds for relying on DNs were satisfactory. Regardless of the role of the FP, conditions for medical treatment were judged by the FPs to be good enough when the grounds for relying on the DN were satisfactory and problematic when they were not.

**Conclusions:**

In the role of conductor, the FP will identify when the grounds for relying on the DN are unsatisfactory and be able to take action, but in the role of consultant the FP will not detect this, leaving home care patients without appropriate support. Only when there are satisfactory grounds for relying on the DN, will conditions for providing home care medical treatment be good enough when the FP adopts a consultative role.

## Background

The shift from hospital to community and primary care for the ageing population has increased the need for all types of home care in Sweden as well as in most western countries [1-4]. Patients with home care are typically elderly people with multiple diseases, a variety of symptoms and reduced functional ability [5-7]. There are three different forms of home care in Sweden. Home help services are social care by home help staff without medical training, Home care by district nurses, is regular care at home by nurses and finally Hospital at home is team based multi-professional health care at home.

A growing number of patients are receiving home health care in Sweden today. In 2008 approximately 200,000 patients received home care, the majority of them from district nurses (DNs). Also, more advanced tasks are being performed, putting more strain on resources. A shortage of nurses and physicians available for this care in Sweden has resulted in a gap between resources and demands that might jeopardise good quality care and home care patients' safety [3,8,9].

An interview study was conducted with Swedish FPs to enhance the knowledge of family physicians' (FPs') experiences with providing medical treatment for patients with home care by DNs. A previous article on this study described the family physicians' efforts to stay in charge of the medical treatment [10]. The patient's problems challenged the FPs' ability to stay in charge, which is why the FP had to especially rely on and collaborate with the DN for the medical treatment of those patients. The present article, based on the same study, further explores FPs' experiences collaborating with DNs when providing medical treatment for patients with home care by DNs.

The need for collaboration between the FP and the nurses concerning home care patients, especially older patients, patients with home care and dying patients, has been identified elsewhere [11-13] and ongoing collaboration between the FP and the nurses in home care and in palliative care at home has been identified [14,15]. Yet other studies found obstacles to developing collaboration such as the remuneration system, differences in medical practice and service delivery and lack of knowledge concerning existing services [11,16]. Integrating physician's services in the home takes time, money and sustained commitment [17]. FPs and DNs may have different views on how to manage matters and how to cooperate [18-20]. The integration of the previously more independently working DNs in the multi-professional HCs and the need for more advanced medical tasks in home care, generating a shift from preventive measures to medical care and treatment, have resulted in a shift of power from the DNs to the FPs. The FPs' use of power in everyday work has been criticised by DNs, putting the issue of collaboration at stake [3,21-23].

The present article concerns FPs' medical treatment in home care by DNs. The term home care by DN was chosen as the most appropriate description as there is no clear definition of this form of home health care in Sweden and there is also a lack of established criteria. In addition, the organisation and resources available for home care by DNs vary extensively both locally and nationally [3]. In the following, home care by DN is referred to as 'home care' and 'home care patients'.

The FPs involved in home care by DNs work in primary care health centres (HC) and are responsible for the medical treatment of all patients listed with them, including patients with home care by DNs and home visits. Home visits by the FP are typically initiated by the DN but may also be initiated by the physician, or on request of the patient or the family. At some HCs, patients with home care by DNs are encouraged to list with one or two FPs who then become responsible for most or all patients with home care, while at other HCs the patients remain listed with the same FP as before. The requirement that physicians employed in primary care must be specialised in family medicine has recently been taken away. Today physicians in other specialities can also be employed. However, in this study only physicians specialised in family medicine employed at an HC were interviewed [9,24].

Health care in Sweden has been subject to competition and budgetary incentives to enhance productivity, increasing the number of patients per day [25]. The demand that patients should be able to see their FP without delay has led to a shift from 'planned' to 'on-demand' care in Swedish primary care. The result is that many FPs no longer initiate regular check-ups for patients with chronic diseases. Instead patients initiate check-ups when they want help [26,27]. However, many home care patients cannot manage this due to reduced functional ability [10].

In home care by DNs the DN is responsible for nursing care. Swedish DNs are licensed nurses specialised in primary care nursing with a comprehensive, psychosocial care perspective. They are responsible for coordinating and directing health services in a geographic district [21,28]. Historically they worked independently in the district but later they were integrated into the multi-professional HCs [21,22]. Nurses without specialised training as DNs also work in home care, as do assistant nurses working under the supervision of a nurse. They will all be referred to as 'DN' in this article. The DNs may work at the same HC as the FPs (the responsibility of the county council) or at a home care organisation (the responsibility of the municipality). Thus there are different conditions for collaboration between FPs and DNs depending on the type of organisation (Table 1) [3,24]. The DNs employed by HCs may work exclusively with home care or have home care as part of their daily work. When the municipalities are responsible for home care by DNs there are large differences as to how collaboration between the DNs and the FPs is organised [3,9,24]. Home care by DNs may be initiated by the FP, the DN or others. Home help services, including social care, are always the responsibility of the municipalities.

**Table 1:** Home care by district nurses in Sweden

Type of responsibility	Type of profession	Responsible authority	Type of organisation
Medical treatment ^1^	Family physician^2^	County Council	Health centre

Home care by^3 ^district nurses	District nurses^4^	County Council or Municipalities^5^	Health centre Home care org.

Home help service	Home help staff	Municipalities	Home care org.

Home care by district nurses is part of primary care but the organisation and which authority is responsible for home care by DNs differ depending on what the local County Council or the Municipalities have agreed on. This will influence the working conditions of the family physician and the district nurse.

^1 ^Including home visits if necessary

^2 ^Until recently, and at the time of the study, physicians in primary care were required to be specialised in family medicine. In reality, many doctors are substitutes without this speciality

^3 ^Differs from hospital at home in that it is not team based. The patient's various care providers handle their part of the treatment as a part of outpatient care. Sometimes, as in the case of the district nurses, it is performed in the home of the patient.

^4 ^An increasing number of the nurses in home care are not specialised district nurses.

^5 ^In more than half of Sweden the municipalities are now responsible for home care by district nurses

Collaboration between FPs and DNs in home care is important but not uncomplicated. The aim of this article was to develop a model to illuminate the process of collaboration between FPs and DNs in the medical treatment of patients with home care as experienced by the FPs, and also to clarify factors that influence this process.

## Methods

Grounded Theory Methodology (GTM) was used to explore FPs' experiences of providing medical treatment for patients with home care by DNs. GTM was chosen because it is a method for studying social processes in areas where little is known [29-32]. The interviews revealed two main concerns that were intertwined in the narratives. In order to separate the processes and be able to describe them in an understandable way the analysis was divided in two when this became clear. One analysis focused on the FPs' problems with staying in charge of the medical treatment due to the patient's problems, and how these problems were managed [10]. The analysis presented in this article focuses on the collaboration between FPs and DNs [31].

### Participants

The setting was primary care in Sweden. FPs were asked to talk about a patient listed with them who had home care provided by DNs.

Thirteen FPs working in a city centre and in suburban, but not rural, areas were interviewed; seven men and six women, ranging in age from 36 to 58. They had worked between one and 20 years as specialised FPs, and between six months and 13 years at their present HC. Both private HCs and HCs run by a county council were included. All FPs were employed at the HC with fixed salaries. Nine worked at HCs that were run by the county councils and four worked in HCs that were privately owned. Of the latter, one was run by a corporate group that owned several HCs, three were run by one or more of the physicians or nurses working at the HC. One of the interviewed FPs was one of the managers of the HC where he worked. All but three FPs worked in the same organisation as the DNs. The number of FP and DN positions at the 13 HCs varied (4-15 FPs and 2-8 DNs), as did the proportion of FP and DN positions filled by FPs and DNs. Five had all FP positions filled and four had one third or more unfilled positions. Eight had all DN positions filled and two had one third unfilled posts or more. In two HCs there was a shortage of both FPs and DNs. This was the situation at the time of the interview, but change over time was also reported. The number of home care patients per FP also varied (Table 2). In two HCs some of the FPs were responsible for all home care patients. Work with home care patients thus made up a substantial part of these doctors' day to day work. The sample of patients is described in table 3.

**Table 2:** Patients receiving home care by district nurses registered with the interviewed family physicians

Label following quotes	Number of family physicians	Number of registered patients with home care	Special home care family physician
A	2	50-60	Yes
B	5	20-35	No
C	4	<10	No
D	2	Did not know	No
	Σ 13		

**Table 3:** The sample of patients included in this study

	Age	Sex	Medical, functional and other problems encountered
1	-	Female	Depression, Pain, Overuse of painkillers
2	61	Male	Alcohol abuse, Epilepsy, Dementia
3	>75	Female	Dementia, Pain, Epilepsy?
4	78	Female	Depression, Dementia and Aphasia after stroke, Incontinence
5	82	Male	Impaired peripheral circulation, Ulcers, Pain
6	85	Female	Asthma, Diabetes, Dementia, Infections
7	86	Male	Prostate hypertrophy, Uraemia
8	87	Female	Dementia, Heart failure, Incontinence, Diabetes
9	87	Male	Diabetes, Obesity, Neuropathy, Both legs amputated, Ulcers, Infections, Pain
10	87	Male	Metastasised kidney cancer, End of life care
11	89	Female	Glaucoma, Bad eyesight, Aortic stenosis, Dizziness and falls, Fractures
12	89	Female	Severe anaemia, Leg ulcers
13	90	Male	Diabetes, Arthrosis, Heart failure, Spanish speaking
14	95	Female	Aged, Deteriorating health, Pneumonia, End of life care
15	Very old	Male	Aged, Heart failure, Angina, Prostate hypertrophy, Dizziness and falls

### Theoretical sampling

The sampling was conducted as a theoretical sampling in accordance with GTM [29], i.e., data were collected continuously and in interaction with data analyses. For a first sample, we asked three FPs at different HCs if they or one of the other FPs at the HC would agree to participate in an individual interview and talk about one of their patients with home care by DNs. FPs were asked to talk about a patient listed with them. They were asked to choose the last patient, aged 65 or older and living in ordinary housing, where they had made a note in the medical record and who met the criteria of having home care by DNs. As many FPs did not have information concerning which of the patients listed with them had home care by DNs, it was not possible to choose a patient that way. Therefore, in interviews 4-12, the FPs were asked to talk about a memorable home care patient, aged 65 or older, in whose care they had been involved. The FPs were much more involved and updated concerning the medical care of these patients. Age was omitted as a selection criterion in interviews 6-13 as the analyses showed that age in itself did not seem important but rather the complexity of the patients' problems, in combination with reduced functional ability, which also was the reason for home care [10]. For the second sample, 24 FPs in one city were invited by letter and asked if they would participate in an individual interview on one of their patients with home care by DNs. Eight answered the letter and agreed to participate. We do not know why the others did not answer. In the first two samples all but one FP worked in the same organisation as the DN. Thus, for the third sample two FPs from a city where the FPs and the DNs typically work in different organisations were selected, both of whom agreed to participate. In all, 13 interviews were conducted. To obtain variation, the FP in interview 13 was again asked to talk about the last home care patient in whose medical record he had made a note. After 13 interviews the research team judged by the analyses that saturation was reached, the material was rich and the same type of answers came up. However, in GTM the judgement of saturation typically includes a certain amount of subjectivity.

### Data collection

Before each interview the project was presented by letter. Face-to-face semi-structured interviews lasting 45-90 minutes were conducted in the FP's office at the HC by the first author who issued the invitations. Informed consent was secured. FPs were asked to give a description of the last time they made a note in the medical record of the selected patient, what problems the patient had and how they were managed. The FP was questioned about the medical problems of this particular patient and how they were managed, which care providers participated and who initiated what was being done. This made it possible to make an assessment of the FP's experience of providing medical treatment for patients with home care by DNs.

*The initial interview guide *is presented below. It was used in the first interview and continually changed to include issues that came up during the interviews. The FP's experience of different problems/situations, in the patient's care, were pursued in the interviews.

The opening question of the interview was: *I would like you to start by giving a short description of the patient and then tell me about the last time you made a note in the patient's record*. Follow-up questions were: *How long have you known the patient; How did you first meet; What are the patient's problems and how are they handled; Do you know anything about the patient's relatives; Do you have any contact with the patient's relatives?*

Further follow-up questions were asked related to each problem/situation that came up: *What does the patient manage on her own; What part of the patient's care do you handle; What other care providers participate in the care; What do they do; Who is responsible for what is done? *Closing questions were: *Is there anything you would have wanted to be different; Is there anything you would like to add? *All through the interview the interviewer focused on the physician's perception of the patient's care and treatment, their experience concerning who was responsible in concrete situations and also who did what on whose initiative.

Each individual interview was analysed before the next interview was conducted, and the interview guide was changed to cover the issues that had emerged during the previous interviews. This way, the experience of the FPs, rather than the preconception of the interviewer, determined the development of the interview guide. The collaboration with the DN appeared as a central issue from the very first interview and was found to be one of the main concerns of the FPs in the following interviews as well. Many different care providers were involved in the care, such as family and friends and other physicians. However, the DNs were the most central care providers in the narratives and several FPs said that without the DNs it would be problematic to manage the treatment of these patients. During the interviews the FP was asked about the problems of this particular patient, how they were managed and also about other professionlas (and relatives) taking part in the care. Follow up questions like what was actually done, by whom it was done and on whose initiative were asked to gather information concerning the FP's experience of collaboration. Collaboration with the DN was central in most of the problems and situations described. The first interviewed FP gave a thorough description of routines for meetings, communication and exchange of information between the DN and the FP. Therefore follow up questions concerning this matter were included in the developed interview guide for the next interviews. During the interview, many FPs consulted the patient's medical and nursing records. Two FPs chose to talk about two patients in order to exemplify conditions they found problematic. Thus 15 patients were included.

Memos were written directly after each interview and during the analyses. The interviews were audio-taped and transcribed verbatim. The transcripts were analysed before the next interview to identify important issues, questions and ideas about links between emerging codes. The memos were used to modify the interview guide and served as a basis for the analysis.

### Data analyses

Based on the GTM method, open, axial (theoretical) and selective coding were performed to enable the emerging theoretical model to be grounded in data. In the coding process the transcribed interviews were read and coded line by line to identify the different factors described by the FPs in the process of collaborating and relying on the DN for home care medical treatment. This included how they acted, saw and experienced the process. Codes were generated to define different factors in the process, formulated in words used by the FPs. Through constant comparison, similar codes were detected and labelled. Data were read repeatedly to find variations and to ensure that the codes were grounded in data. Codes with the same meaning were grouped in descriptive categories and the theoretical properties of the categories were generated through comparison. The categories were sorted into higher-order categories and subcategories, which were subsequently compared with one another to form concepts such as *conductor *or *consultant*. Constant comparisons were carried out until saturation was judged to be attained. Axial coding was used to identify how the different concepts were related to each other. Patterns were analysed and a core process emerged through selective coding linking all the concepts to the core category, i.e., *to rely on the DN in their medical treatment of home care patients*.

All the authors participated in the analysis. The team was comprised of people with different backgrounds (two FPs, a DN, a psychologist and a researcher specialising in GTM) to bring a variety of knowledge to the process. Open coding was mainly done by the first author (SM), who is an FP. The team members monitored the progress and expressed their views. The other researchers actively participated in the axial and selective coding process. The computer program NVivo was used. Quotes are used to illustrate the findings. The number at the end of each quote is related to factors about the patients, presented in table 3, and the letter is related to factors about the FPs, presented in table 2. The project was approved by the Research Ethics Committee South, Karolinska Institute, Stockholm.

## Results

The result is a model illuminating the core process of 'how FPs rely on DNs in home care' medical treatment by describing how FPs retain or leave the initiative to the DNs.

In the presentation of the results the following are described:

• the concept of 'rely on',

• factors influencing whether FPs retain or leave the initiative in the medical treatment to the DNs,

• two different types of roles that FPs adopt, *the medical conductor *and *the medical consultant*, characterised by how much initiative FPs retain,

• factors influencing the FP's experience of satisfactory grounds for relying on the DNs for medical treatment,

• in what circumstances conditions for medical treatment are considered good enough or problematic in relation to the role taken by the FP and in relation to the grounds for relying on the DN for medical treatment (satisfactory/not satisfactory).

### Relying on DNs in home care

Home care for these patients was initiated by the DN, the FP or the FP and DN together and also by the hospital after inpatient care. Most FPs were very satisfied with the collaboration with the DNs, especially if they had worked together for a long time, which was common. The FPs described it as 'fundamental' to be able to rely on the DNs in home care medical treatment. All felt that they as FPs needed to have contact with the DNs, sometimes frequently, and that they needed easy access to the DNs through regular meetings, on-demand meetings or telephone calls.

'I feel that you have to have a competent district nurse that you can rely on, someone who tells you how things are, that you can go through them (the patients with home care)'. 3B

Good home care was seen as a basis for providing medical treatment. The FPs expected the DNs to assess the need for and provide necessary home care, manage contacts and meet with family and neighbours, home help staff and hospitals to coordinate care and treatment.

*'I really feel that it is the district nurse that should be the organiser' *5A.

In addition to regular home care the FPs *relied on the DN for medical treatment in home care *(written as 'rely on'). Rely on meant for the FP to trust the DN to carry out investigations and treatment initiated by the FP, to inform the FP and mediate contact with patients when asked.

'*Home care patients with diabetes need to be checked. They (the district nurses) must look at the feet regularly, once a month, and check the blood sugar at home once a month*' 8B

However, rely on also meant that the FP trusted the DN to mediate contact and information between them and the other care providers. In addition, the DN mediated contact between them and the patient on her own initiative, and alerted the FP when unexpected problems appeared, e.g., if a patient's health deteriorated or if a patient did not take the medicine.

*'Well, the district nurse is there every day, she gives insulin every day; if something happens I get alerted directly when she calls'*, 9D

*'The district nurse had seen her at home before and so she told me a bit. No, they booked a visit for her so she came with the home help staff. They had told me about her problems in advance when they booked the visit' *4B

Rely on also meant that FPs trusted DNs to take the initiative to send a patient to hospital in the event of accidents or other emergency situations.

Some FPs relied heavily on the DN's initiative, while others retained more of the initiative as described below under the heading of the role of the FP.

### Factors that influence how much of the initiative the FPs retain

Factors that influenced whether FPs retained or left the initiative to the DNs were the patient's *disease*, the FPs' *attitude *and the FPs' *working conditions *(Table 4). Sometimes FPs left the initiative to the DNs because they found it satisfactory, but sometimes they had to leave more of the initiative to the DNs than they found satisfactory because of their working conditions.

**Table 4:** Factors that influence whether family physicians take the role of conductor or consultant

Conductor (retains initiative for medical treatment)	Consultant (leaves initiative for medical treatment to the district nurse(DN))
• Working conditions: FPs' working conditions are good enough • Attitude: FPs have a positive attitude towards being a conductor • Disease: The type of disease in need of FP initiative	*FPs feel forced to take a role as consultant:* • Working conditions: FPs' working conditions are problematic • Attitude: FPs have a positive attitude towards being a conductor instead of a consultant
	
	*FPs choose to take a role as consultant* • Attitude: FPs have a positive attitude towards being a consultant • Disease: The type of disease not in need of FP initiative

#### The disease

The type and stage of the disease influenced how much initiative FPs left to the DNs. The same FP could retain the initiative; plan future visits, investigation and treatment of one type of disease; and leave the initiative to the DNs for the treatment of another type of disease that the FP relied on that she could manage independently. One example is the FP who retained the initiative concerning a patient's recurring anaemia but relied on the initiative of the DN for the treatment of the same patient's leg ulcers 12C. Further examples of diseases left to the DN's initiative were malnutrition 8B, incontinence 8B, and diabetes checkups 13C. Circulatory diseases 5A, 12C, 8B, cancer treatment 10A and pain control 5A, 9D, on the other hand, were examples of diseases managed by the FP.

#### The FP's attitude

The attitude of the individual FP influenced how much initiative the FP left to the DN. Some FPs retained the initiative and planned future visits for chronic diseases. Others had adopted on-demand care and expected patients to initiate all FP visits, even follow-up visits for chronic diseases. It was natural for FPs to rely on the initiative of the DNs if these patients could not manage to do this.

'*When they cannot look after themselves they become home care patients. Until then they look after themselves, we do not send appointment notifications*' 15B.

Another FP expressed some doubts about the attitude he had adopted.

*With these patients, you often have a defensive role as family physician, you try to handle problems that others signal exist, and do not have a strategy of your own for the treatment but handle the problems as they appear, contrary to other patients with chronic problems where you have an idea about how to control and treat 11C*.

#### The FPs' working conditions

Insufficient time or routines at the HC and organisational boundaries were working conditions that could prevent FPs from retaining the initiative. They could force FPs to leave more of the initiative to the DNs than they found satisfactory.

##### Insufficient time

Reasons for *insufficient time for medical treatment in home care *included heavy work load due to too many patients, shortage of staff among FPs (vacancies or too few positions) and demands for many visits per day. If there was insufficient time for meetings with the DNs they became too few and too short. Matters had to be managed during breaks or between visits. There was only time for urgent medical problems and matters and no time to discuss future care and treatment. Home visits were time consuming and therefore hard to perform, so FPs had to leave more of the initiative to the DNs who could make home visits.

*'You have insufficient time to familiarise yourself with a case, you just have to manage the problem of the day and try to get sufficient information to know that you're doing the right thing at the moment. Yes, sometimes things are done twice, and yes, sometimes things are missed*' 12C.

##### Routines at HCs

Several FPs did not have information about which of the patients listed with them had home care. This meant that it was difficult to retain the initiative. At some HCs there was no notification in the records, at other HCs the records of the home care patients were marked, but many FPs did not have the assembled information that the DNs had about which of their patients had home care. Some FPs had regular meetings with the DNs for interprofessional consultations and exchange of information concerning all home care patients. Others had only on-demand contact with the DNs. *'Not regular contacts like every week or so. They visit her at home, so it is more that they (the DNs) contact me when they think there is a need' *12C. FPs' preparations before each home visit were time consuming, unless there were routines to support home visits. As the FPs were usually fully booked, it was difficult to manage emergency home visits.

##### Organisational boundaries

One reason FPs did not know which of their patients had home care was that FPs and DNs worked in different organisations. FPs were not always informed when home care was started for a patient and had no access to the nursing records. Reorganisations could mean, e.g., that they did not know which DN to contact or whether they or the physicians in the special home care organisation were medically responsible for a patient. This caused problems when the FPs referred patients to the hospital and they were referred back to the home care organisation. *'We are still the physician in charge even though we really don't know' *7D. In all these circumstances, it was difficult or impossible for the FP to retain the initiative.

### The role of the FP

Thus the patient's disease, the FP's attitude and the FP's working conditions influenced how much initiative the FPs retained or left to the DNs. On the basis of the interaction of these factors, two different roles could be identified; the *medical conductor *and the *medical consultant*, here called conductor and consultant. The roles are thus characterised by how the FPs retain or leave the initiative to the DNs, which in turn depends on the patient's disease, the FP's attitude and the FP's working conditions. The individual FP could adopt the role of conductor for one disease and consultant for another. The individual FP could also adopt some of the features of the two roles. FPs could choose to adopt the role of conductor or consultant, but working conditions could also force them take the role of consultant in situations when they did not find it satisfactory to do so.

#### The medical conductor

Taking the role of conductor means that FPs retain the initiative. Conductors plan the medical treatment and future visits for patients with chronic diseases and contact the DNs for collaboration. The conductors felt that they had an extra responsibility for home care patients and that it was important to know who these patients were. Conductors get more involved when patients have home care by DNs as compared to other patients.

As conductors the FPs saw these patients as often as other patients and had frequent interprofessional consultations with the DNs. '*Home care - well, this doesn't reduce my contacts but... no, it doesn't. On the contrary, the contact increases - I get more involved*' 6B.

They were distressed when they did not know which of the patients listed with them had home care. They regarded home visits as a special opportunity to gain information and contact, initiate care planning, and they did not always rely on the DN's coordination. They could introduce new routines when old ones were insufficient. They also initiated joint activities with the DNs, planned home visits, performed them together with the DNs and initiated interprofessional consultations where they asked for information, discussed problems and made joint decisions.

*'I try, if it is a chronic patient that I know I will treat, I usually initiate a care plan. If you can call it care planning, at least a dialogue with those who are involved/.../I find that very important. It's like making a first examination of the patient*' 10A.

#### The medical consultant

Taking the role of consultant means leaving the initiative to the DNs. Consultants rely on DNs to arrange future FP visits for both acute conditions and chronic diseases and to manage some diseases independently, contacting the FPs when necessary. Consultants do not experience any special responsibility for home care patients and do not feel a need to know who they are. Consultants think they can see patients who have help from the DNs less often than patients with no home care by DNs. '*So I have seen him twice in a year. I think that is sufficient when you have such good contact with him through the nurse*' 13C.

Consultant FPs did not reflect on, and were not bothered by, not knowing which of their patients had home care. They did not express a need for home visits, or feel that a home visit would make a difference. Several FPs had never made a home visit to the patients they talked about. They saw the patients during visits to the HC and relied on the DNs to arrange these visits at an agreed frequency, such as once a year for diabetes.

Consultant FPs left the initiative for joint home visits or joint visits at the HC to the DNs, e.g., when she wanted a medical assessment. *'She was taking antibiotics and they (the DNs) wondered if the treatment should continue' *12C. They also left the initiative to provide information and have discussions during interprofessional consultations to the DNs, e.g., concerning discussions about the effect of treatment. They expected the DNs to contact them when they needed medical advice or support. Support of the DNs could sometimes be the FP's most important task in home care. *'I am really a sort of support for the DNs, you could say' *2C. Independent medical treatment by the DNs could be, e.g., to manage incontinence or to send the patient to hospital in an emergency situation.

### Factors that influence FPs' experiences of satisfactory grounds for relying on the DNs

Most FPs felt that there were satisfactory grounds for relying on the DNs, but there were exceptions. When working conditions *forced *FPs to act as consultants they had to rely on the initiative of the DNs sometimes more than they found satisfactory. According to the FPs three factors were important in deciding whether or not satisfactory grounds for relying on the DNs in the medical treatment existed: *the disease*, *the DNs' attitudes *towards collaboration with the FPs and *the DNs' working conditions *(Table 5).

**Table 5:** Factors that influence the grounds for FPs to rely on DNs

Satisfactory grounds for relying on the DN	Unsatisfactory grounds for relying on the DN
• Working conditions: DNs' working conditions are good enough	• Working conditions: DNs' working conditions are problematic
• Attitude: DNs have a positive attitude towards collaborating with FP	• Attitude: DNs do not have a positive attitude towards collaborating
• Disease: Type of disease that DNs can manage	• Disease: Type of disease that DNs have problems handling

#### The disease

The type and stage of a disease influenced whether or not satisfactory grounds existed for relying on the DNs for independent medical treatment. Nutritional problems that did not require nutritional beverages 8B, ulcers where a circulation test had been carried out 12C, problems with incontinence 8B and follow-up of diabetes between yearly FP visits 13C were mentioned as examples of problems that could be managed independently by the DNs.

#### The DNs' attitudes

DNs' attitudes towards collaboration influenced whether there were satisfactory grounds for relying on the DNs to provide information and involve FPs when necessary. FPs and DNs did not always agree on when the FP should be involved or how a problem should be solved. Regular meetings did not guarantee that the DNs discussed matters the FPs found important. FPs thought that they did not always get sufficient information concerning such things as prescriptions or the patients' medical conditions.

'*The intention is that we should be able to make joint plans when we see each other at those rounds, e.g., if nutritional beverages are needed, then it is...now she has prescribed it herself but usually the physician prescribes this/.../I feel that nutritional beverage is the wrong solution (for patients with dementia*)' 8B.

'*I sort of sat here with a lot of notes and prescribed a lot of medicines but I was not sure if they were my patients or not. It was sort of just a job that had to be done because the DNs had to fill the dose dispensers*' 1B.

#### The DNs' working conditions

Two different factors in the DNs' working conditions influenced whether or not satisfactory grounds for relying on the DNs in the medical treatment existed: if the DNs had *insufficient time *or were *short-term substitutes*.

*Insufficient time *was due to shortages of DNs to fill the posts or too few posts. Time for interprofessional consultations was limited. The DNs did not have time to provide sufficient information, e.g., in connection with requests for prescriptions. One FP reported that he wanted to know how the patient took a medicine and if it helped, but could often not get this information *'The DNs are so stressed that they forget their profession. They just take orders' *5A. Insufficient time could lead to mistakes and errors, but FPs hesitated to report such errors knowing that lack of time was the reason. *'Sometimes you find out that even though we had agreed on a medicine, two weeks later the patient still did not get it' *5A. Several FPs worked with DNs who were *short-term substitutes*, who might not have the same formal training as a DN. They had less information to provide as they did not know the patients and their problems. The information might even be incorrect. Short-term substitutes might have a short-term attitude about how to manage problems.

'...*has a different approach than someone who knows that she will stay longer, so there can be poor - what can I say - poor empathy with the patient's problems, so to speak. It's more about solving the problems of the day*' 5A.

### Relying on the DN for medical treatment - an integrated model

According to the analyses, conditions for providing medical treatment in home care by DNs is related both to the role of the FPs and to the grounds for relying on the DNs (satisfactory/non-satisfactory). The integrated model is described in table 6. According to this model conditions for providing medical treatment in home care are good enough when there are satisfactory grounds for relying on the DN in the medical treatment irrespective of what role the FP chooses or is forced to take. Likewise the conditions for providing medical treatment are problematic when there are unsatisfactory grounds for relying on the DN in medical treatment irrespective of the role of the FP. However, if the problematic situation is detected and how it is managed depend on the role of the FP.

**Table 6:** Good-enough or problematic conditions for medical treatment

	Satisfactory grounds for relying on the DNs	Unsatisfactory grounds for relying on the DNs
**FPs take the role of conductor**	**a**.	**c**.
	Good enough conditions for medical treatment	Problematic conditions for medical treatment. FPs use strategies to overcome the problems
**FPs take the role of consultant**	**b**.	**d**.
1. FPs feel forced to take the role of consultant	b1. Good enough conditions for medical treatment	d1. Problematic conditions for medical treatment. FPs use strategies to transform their role from consultant to conductor
2. FPs choose to take the role of consultant	b2. Good enough conditions for medical treatment	d2. Problematic conditions for medical treatment. FPs not aware of problems

Conductor FPs are aware of the problems and use various strategies to overcome them. In our study the FPs demanded information, initiated regular meetings and demanded more time for meetings. Forced by working conditions to be a consultant, the FPs use various strategies to transform their role from consultant to conductor. One FP described how he managed to get the list of home care patients he was responsible for by first asking the DN and finally going to the head nurse 1B. FPs also used lunch breaks and unexpected free time to be able to make home visits and to familiarise themselves with the patients' problems. They also established their own priorities, their own routines and bent the rules when necessary.

'*I have decided on my own that I will begin to make home visits to them (the home care patients). We are supposed to see at least ten patients a day. That is not possible on a day when we go to someone's home and I have scheduled a meeting with the district nurse*' 1B.

*'Yes I can do it (make emergency home visits) in connection with lunch' *15B.

However, if FPs choose to be consultants in situations when there are unsatisfactory grounds for relying on the DNs in the medical treatment the consultant will not be able to identify the situation as problematic as the DNs will not provide the FP with information, or involve the FP in the care and treatment. The consultant has no strategies to overcome unknown problems. It turned out that the FPs were not aware of this type of situation; they discovered it as they went through the nursing records during the interview.

Thus, we have identified three types of problematic conditions due to the type of collaboration between the FP and the DN. Two of them are conditions when the FP will use strategies to try to overcome or compensate for the problematic condition and one in which the FP is not aware of the problems and thus will not try to overcome or compensate for them.

## Discussion

### Short summary

In home care described here, it is fundamental for FPs to be able to rely on the DNs for the medical treatment, but the disease, FPs' attitudes and FPs' working conditions influence how much of the medical treatment initiative FPs retain or leave to the DNs. Two roles have been identified - the medical conductor who retains the initiative, and the medical consultant who leaves the initiative to the DNs. The individual FP can be a conductor for one disease and consultant for another. Depending on the disease, DNs' attitudes and DNs' working conditions, the grounds for relying on the DNs may be perceived as satisfactory or not. FPs' working conditions can also force them to leave more of the initiative to the DN than they find satisfactory. Conditions for medical treatment are described as good enough when there are satisfactory grounds for relying on the DN and as problematic when they are unsatisfactory, regardless of the role of the FPs. When conditions are problematic, FP conductors use various strategies to change this. FPs as consultants, however, are not aware of problematic conditions for medical treatment as they do not get information and are not alerted to problems when there are unsatisfactory grounds for relying on the DNs in the medical treatment.

### Conductor/Consultant

The role of conductor or consultant was taken either by choice or by necessity due to working conditions. Although official documents and goals seem to advocate a role as conductor, in practice this is not always the case. WONCA Europe, defines the core competencies and characteristics of family medicine as: responsibility for provision of longitudinal continuity of care as determined by the needs of the patient and for making efficient use of health care resources through coordinating care and working with other professionals in the primary care setting [33]. In reference to the terms of the present article this is clearly a description of a conductor rather than a consultant. The present study shows, however, that being a conductor is problematic when not having information about which of the listed patients have home care. Furthermore, patients like those in home care [5] should be a top priority according to priority discussions in Sweden [34]. However, this study shows that insufficient time made it difficult to be a conductor, which corresponds to a recent survey of home care in Sweden that revealed increased workload in combination with shortages of physicians and nurses [3]. The shift from secondary to primary care has also resulted in more advanced measures in home care by DNs and thus a greater need for FP involvement in this form of care, which the DNs used to manage independently. The traditional way of working may certainly have influenced the attitude of both the FPs and the DNs in such a way that the FP continues to rely on the initiative of the DN even when the type or stage of disease is so severe that it is not satisfactory to do so [3]. These recent changes may also account for why the HC has not yet created sufficient routines, e.g., the lack of a system for providing the FPs with information about which of their patients had home care.

The conditions for home care by DNs and collaboration between DNs and FPs in this care have changed drastically in Sweden. New health care reforms have introduced new organisational boundaries and thus made collaboration between FPs and DNs more difficult. Also budgetary incentives have made many HCs prioritise practice visits over home visits [25,35].

These reforms have also resulted in a shift from 'planned' to 'on-demand' care [26,27] which may have paved the way for FPs as consultants. When home care patients no longer manage to contact their FPs [10], the initiative is transferred to the DNs. Being a consultant in the situation when there are satisfactory grounds for relying on the DNs could be one way to make efficient use of health care resources [33]. When the DN is involved it may be sufficient to see patients less often and leave more of the initiative to the DNs. However, in order to take the role of consultants FPs need to know if the DNs' work situations are such that satisfactory grounds exist for relying on them. We found that the role taken by the different FPs varied considerably in part due to a difference in attitude of the individual FPs as well as the diseases of the individual patient. How the patients, the DNs, and the other care providers experience the FPs' attitude and role taking can not be answered by this study interviewing the FPs themselves, but requires another type of study.

### Rely on

The majority of the FPs in our study were satisfied with the collaboration and felt they could rely on the DNs. They said that the DNs acted as intermediaries between FPs and patients, and between FPs and family and home help. The intermediary role of the DN has been emphasised [17,28]. Many studies identify that there is a need for FP - DN collaboration for patients with home care in order to enhance the care and treatment of older patients and patients with palliative care [10-12]. The FPs also managed the increased workload due to the shift from secondary to primary care by shifting tasks to the nurses [36]. However, the concept of relying on DNs in medical treatment has not been explored to our knowledge.

FPs' working conditions influenced how they relied on the DNs in the present study, which is in accordance with earlier studies about how care is organised and financed [3,37,38]. Also, the DNs' working conditions were crucial for whether or not there were satisfactory grounds for relying on the DNs. In this qualitative study we identified some factors that influence this according to the FPs. To identify the extent of this influence other studies with a more quantitative design must be performed. Since an increased workload in combination with a shortage of home care nurses has been detected, this needs further study as the role of the DN in this form of home care is essential[3,10]. Several FPs talked about, e.g., nurses who were short-term substitutes and lacked a DN's formal training as one reason for unsatisfactory grounds for relying on the DNs.

DNs' attitudes towards collaboration were one factor that influenced whether the FP felt that there were satisfactory grounds for relying on the DNs. The attitude towards involving the FP and providing the FP with information may have been influenced by the changed roles of both FPs and DNs in home care. Historically DNs managed home care more independently [21]. Today there is a need for more advanced home care medical treatment [3] and, therefore, for the participation of FPs and for collaboration between FPs and DNs. Being integrated in HCs, the DNs now work closer to the FPs. This has changed the power balance in a way that DNs may see as negative [21,23], and that may negatively influence their attitudes towards collaboration with the FPs. On the other hand, many of the FPs interviewed in this study expressed a positive attitude towards relying on the DNs. Their previous experience of home care as the DNs' field of responsibility may have contributed to this and facilitated a consultative attitude. Also, other studies show that FPs depend on the information and assessments of DNs as they don't have time for it themselves and thus have to trust the DNs [10,23].

For FPs to rely on the DNs in an appropriate way, especially in the role as consultants, the views of FPs and DNs on care have to agree. Different views [39] and lack of interprofessional knowledge about other care providers' strategies [18,19] can negatively influence collaboration. Interdisciplinary team-building exercises, meetings and regular face-to-face contacts were found to be essential for integrating physicians into home care services [17]. Many FPs in our study stated that it was not possible to take time to plan future treatment together; only the most urgent matters could be managed. This meant that an essential factor for knowing each others' expectations and strategies was missing.

Most FPs felt that satisfactory grounds for relying on the DNs existed, inferring that the conditions for providing medical treatment were seen as good enough. However, in some instances the working conditions in today's home care, incompatible attitudes as a result of changed roles, and lack of time for planning together, can undoubtedly contribute to problematic conditions. It is seen as alarming that FPs functioning as consultants were not aware of these problematic conditions. If FPs rely on the initiative of the DNs, unaware of the unsatisfactory grounds for relying on the DN, the home care patients will be left without proper support for their medical treatment.

### Strengths and weaknesses

The strength of our study is that we have been able to distinguish a specific process: 'to rely on', conceptualising the collaboration between the FPs and the DNs from the perspective of the FPs, and to create a model depicting factors that influence the process and the effect of these factors on conditions for providing home care medical treatment. GTM was well suited for studying this social process and creating a relevant model grounded in data [29-32]. As is always the case with GTM, further research is needed to test the relevance in other collaborative situations between FPs and DNs.

The fact that the interviewer (SM) is an FP who interviewed her own peers with whom she had previous contact, is both a weakness and strength. If the interviewer is seen as a peer and confidant, this may facilitate access and result in richer and more personal accounts of attitudes and behaviour, but it can also influence the researcher's ability to obtain data because of shared understanding that is not explored [40,41].

The theoretical sampling procedure contributed to the provision of rich data but also to a lack of data about patients with whom FPs as consultants were less actively involved. Interviews with FPs about *any *patient in home care would have given more information about consultants not knowing about problematic conditions. However, interviewing FPs about situations that are problematic because they are not informed about them is, in itself, problematic. Thus more mixed research methods would have to be used to explore this potentially problematic type of situation.

## Conclusion

In home care medical treatment, FPs sometimes adopt a consultative role, leaving the initiative to the DNs. The result is seen as good enough conditions for providing medical treatment as long as there are satisfactory grounds for relying on the DNs. FPs who retain more of the initiative, taking the role of conductors, detect when conditions for providing medical treatment are problematic because there are unsatisfactory grounds for relying on the DNs and they use various strategies to change the problematic conditions. FPs as consultants, however, will not detect if there are unsatisfactory grounds for relying on DNs. Thus they are not aware of problematic conditions for providing medical treatment, leaving home care patients without sufficient support.

### Practical implications

The collaboration between FPs and DNs in home care medical treatment is an important process as the FPs have to rely on the DNs in order to be able to stay in charge of the medical treatment [10]. Identifying how this collaboration can take place and factors that affect it has practical implications when the development of home care and home care medical treatment are discussed both locally and on a nationel level. It can also help the individual FP and DN to understand more about their collaboration and how they depend on each other, thereby enabling them to better manage the problems they encounter. Our research supports the idea of joint discussions as a means for FPs to make sure that the DNs' working conditions are such that there are satisfactory grounds for relying on them in the medical threatment.

### Implications for future research

It is urgent to study the situation of FPs not being aware of problematic conditions for providing medical treatment with a more multifaceted study design. It would be of interest to explore it also from the perspective of the patients and the DNs, who might have a different perspective than the FP as to whether the attitude and role adopted by the FPs can be seen as good enough. It would also be interesting to explore it simultaneously from the three different perspectives. A study design concerning decision-making, where different FPs have to choose solutions for cases with a variation of different standardised problems/situations in home care, could explore the differences in attitude among FPs. More studies are needed in different contexts to explore how common the factors are that we have identified as influencing collaboration.

## Competing interests

The authors declare that they have no competing interests.

## Authors' contributions

SM and AKF were responsible for the original idea. SM designed the study with some external help, issued all the invitations and conducted all the interviews, and was responsible for the initial analyses. SM wrote the first draft and subsequent revisions of the manuscript under the supervision of IH. All authors have been involved in the data interpretation, in critically revising the manuscript in terms of important intellectual content, and all of them read and approved the final version of the manuscript.

## Pre-publication history

The pre-publication history for this paper can be accessed here:

http://www.biomedcentral.com/1471-2296/11/82/prepub
